# Regional variations in vaccination against COVID-19 in Germany

**DOI:** 10.1371/journal.pone.0296976

**Published:** 2024-04-18

**Authors:** Verena Bade, Hendrik Schmitz, Beatrice Baaba Tawiah

**Affiliations:** 1 Paderborn University, Paderborn, Germany; 2 RWI – Leibniz Institute for Economic Research, Essen, Germany; 3 Leibniz Science Campus Ruhr, Essen, Germany; 4 Munich Research Institute for the Economics of Aging ans SHARE Analyses, Munich, Germany; Universite Paris Pantheon-Assas, FRANCE

## Abstract

Vaccination willingness against COVID-19 is generally perceived as low. Moreover, there is large heterogeneity across and within countries. As a whole, Germany has average vaccination rates compared to other industrialized countries. However, vaccination rates in the 16 different German federal states differ by more than 20 percentage points. We describe variation in vaccination rates on the level of the 400 German counties using data on all vaccinations carried out until December 2022. Around 52-72% of that variation can be explained by regional differences in demographic characteristics, housing, education and political party preferences. We find indications that the remaining part may be due to differences in soft factors such as risk aversion, trust in the German government, trust in science, and beliefs in conspiracy theories regarding the origins of the Corona virus. We conclude that improving the trust in science and the fight against conspiracy theories may possibly be effective tools to improve vaccination rates and effectively fight pandemics.

## Introduction

In order to curb the SARS-CoV-2 virus, vaccines were developed as quickly as possible in 2020 and enormous vaccination campaigns started by the end of 2020 in the industrialized countries. In the first months of 2021, undersupply of vaccines was the major problem of these vaccination campaigns. Yet, this changed throughout the year and, later, too low vaccination willingness was considered the major challenge to yield herd immunity [1–3]. Herd immunity, in turn, was among the most important health policy goals in most countries in order to overcome the Corona crisis. However, vaccination willingness against COVID-19 is generally perceived as low [4–6]. While several mutations of the virus made the original vaccinations less effective—but also reduced the severity of the virus [7]—there is little controversy that vaccination still is considered a highly effective tool to reduce mortality and severe illness after a COVID-19 infection [8, 9]. This holds in particular in comparison with other measures such as lockdowns or school closures which have extremely high social costs [10–13].

Understanding the determinants of vaccination status is necessary to improve the success and acceptance of vaccination campaigns—both for future pandemics and also for endemic viruses such as influenza and potential mutations of the Corona virus [14]—even more so as vaccination hesitancy is not peculiar to just the COVID-19 vaccines but vaccines in general [15, 16]. Another dimension of vaccination hesitancy is the one towards the COVID-19 vaccine booster shots [17–19], which is even stronger compared to the first two doses of COVID-19 vaccines [20]. One of many potentially fruitful ways to study the determinants of vaccination status is to analyze the determinants of regional differences in vaccination rates. Studying regional variations helps in at least three ways. First, it allows to get a benchmark of which vaccination rates might be achievable in a thought experiment where all regions have the same rates as, say, the region with the highest ones, or, for instance, as those in the upper quartile. It also makes transparent how much room there is for improvement, that is, if all regions are fairly similar or some lack behind by large amounts. Second, it enables to study which observable variables account for the regional differences. In principle—although beyond the scope of this paper—one might figure out what regions with low rates may learn from regions with higher rates in order to increase vaccination rates. Yet, not each and every difference between regions is necessary as sign of inefficiencies. Thus, as a third point, the analysis of regional variations may help to understand which degree of differences is acceptable, for instance, because differences in age structure might imply justified differences in vaccination rates.

**Fig 1:**
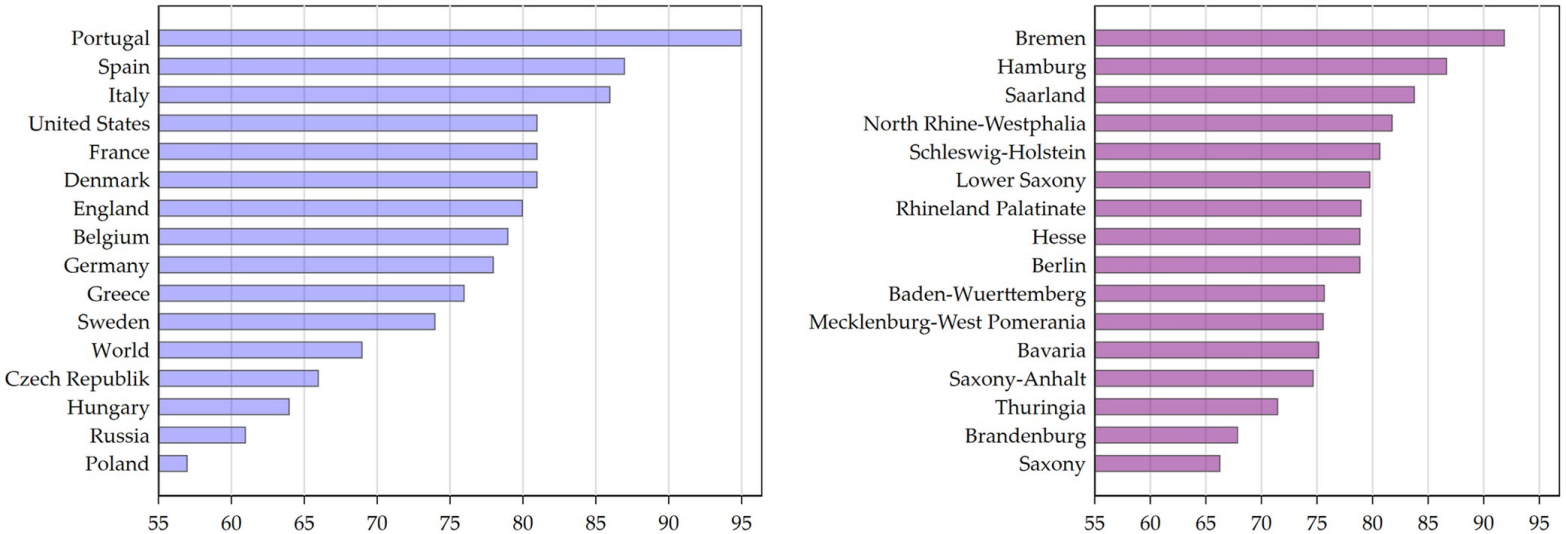
Share of people vaccinated against COVID-19. Vaccinated at least twice as of January 2023. Left panel: Worldwide. *Source:* Our world in data, https://ourworldindata.org/covid-vaccinations; Right panel: German states. *Source:* Robert Koch Institut, https://www.rki.de/DE/Content/InfAZ/N/Neuartiges_Coronavirus/Daten/Impfquoten-Tab.html.

As a point of departure of our analysis, the left panel of Fig 1 reports the share of people with at least two Corona vaccinations (typically considered as those with completed immunization) across selected European countries and the USA. Among the European countries, Germany—the country we study in this paper—is somewhere in the middle with about 78% of its population having completed the initial vaccination protocol, i.e., two vaccination doses for most vaccines. However, only looking inside Germany—as the right panel of Fig 1 does—we see that variation within Germany is almost as large as the one across the countries in the left panel. The right panel shows vaccination rates in the 16 German federal states. For instance, while the share of vaccinated people in Saxony is as low as the mean in the Czech Republic (around 66%), the share in the Saarland (84%) is almost as large as the share in leading European states such as Italy and Spain. With more than 20%-point differences across German regions, it seems important to understand what factors might be associated with these differences. In this paper, we analyze correlations of regional variation in Corona vaccination in Germany. We hypothesize that differences in demographic status, levels of education in the population and economic situation could account for some of these differences. We further hypothesize that political preferences and susceptibility to conspiracy theories also add to regional differences in vaccination rates.

### Background

Fig 1 shows considerable regional variations in COVID-19 vaccine uptake. This has also been found in other countries. Most of the studies on the regional variation in COVID-19 vaccine uptake are based on the USA. [21] find more variation across counties than states and [22] find more variation in vaccination levels across postal codes within cities than between cities. A common source of variations in COVID-19 vaccinations in the US is racial disparities. However, the findings are mixed: [22] find higher vaccination rates in areas with a high share of white or Asian people and [23] find lower vaccination rates in regions with a high share of Black people. These findings differ from the results of [24] who find that minorities, defined as all persons except white, non-Hispanic, have a positive impact on vaccination rates, and from the results of [2] who finds that regions with a high share of non-Hispanic white residents have lower vaccination rates. Political affiliation has also been associated with vaccine uptake, where counties with a large proportion of Republican voters have lower vaccine uptake [2, 23]. Other socio-demographic and socio-economic factors which have also been related with the regional variation in vaccine uptake include the share of population below the poverty line, the share of uninsured persons, the share without internet access and the share of younger persons, where higher shares are associated with lower vaccination rates, and the share of persons with a college degree, the share of older persons and per capita income, where higher levels are associated with higher vaccination rates [2, 21–24].

Some of these factors associated with COVID-19 uptake also correlate with compliance or non-compliance with other COVID-19 preventive measures. Political affiliation is mostly found to be associated with the compliance or non-compliance with COVID-19 preventive measures, where Democrats in the USA are more welcoming to measures such as social distancing [25–27]. Poverty [28] and the share of younger population [29] have been associated with lower compliance with social distancing.

For Germany, [30] find considerable variations in vaccination rates between federal states: states in West Germany have higher vaccination rates than those in East Germany. They also find that support for the populist right-wing political party, Alternative for Germany (AfD), is correlated with lower vaccination rates at the state level. Among the studies done on regional variations in COVID-19 vaccinations, only a few, such as [23] and [24], have tried to explain the variations. All in all, with an adjusted *R*^2^ of about 76–79% these studies can explain a considerable share of variation in vaccination rates in the USA by observable characteristics. Our study contributes to the literature by bringing in a new perspective from not only a different country but a country with a different health care system.

Our paper also adds to a growing field of health economic research on regional variations in the health care market. Strong regional variations have been found in many dimensions across different countries. Differences have been found in health [31], health expenditures [32–38], access to healthcare [39], medical services utilization [40–45], and productivity in the health care sector [46]. These differences may be linked to the variation in vaccine uptake. [23] find that counties with more health facilities have higher vaccination rates. They argue that a lack of these health facilities may have resulted in the delay or loss of opportunity to vaccinate in some regions.

We study regional variations in the share of at least double-vaccinated persons in Germany using administrative data on all vaccinated doses in all 400 German counties until December 2022. Using a host of potentially relevant socio-economic variables (guided from determinants found in the previous literature) we can explain about 52 per cent of regional variations in vaccination on county level and 72 per cent on federal state level. Major explanatory factors are demographic characteristics, levels of education, and further preferences proxied by political party vote shares. To get an idea of what might account for the remaining part, we describe the federal state level distribution in selected “soft” factors and document that individuals in federal states with lower vaccination rates, on average, report to be less risk averse, have less trust in the German government and scientists, and are more likely to believe in conspiracy theories regarding the origins of the Corona virus. While our study design is not able to provide any causal evidence and, thus, does not allow to immediately derive policy recommendations, it adds to our knowledge on the picture of regional variations in health care.

## Methods

### Vaccination rates

Our main dependent variable is the share of individuals with at least two vaccinations against the Corona virus. It is based on freely accessible and continuously updated data from the German Robert Koch Institute (RKI) [47] that publishes data on the universe of daily vaccinated doses per age group on the level of the 400 German counties. German counties can either be rural areas (*Landkreis*, *N* = 293) or single larger cities (*Kreisfreie Stadt*, *N* = 107). The RKI vaccination data are assigned to the county where the vaccination took place and not where the vaccinated individual lives. In principle, individuals can freely choose where to get vaccinated and it may be possible that they get vaccinated in neighbouring counties. In particular, it seems to be frequently done that individuals in rural areas get vaccinations in the next bigger cities where larger vaccination centers had been installed.

**Fig 2:**
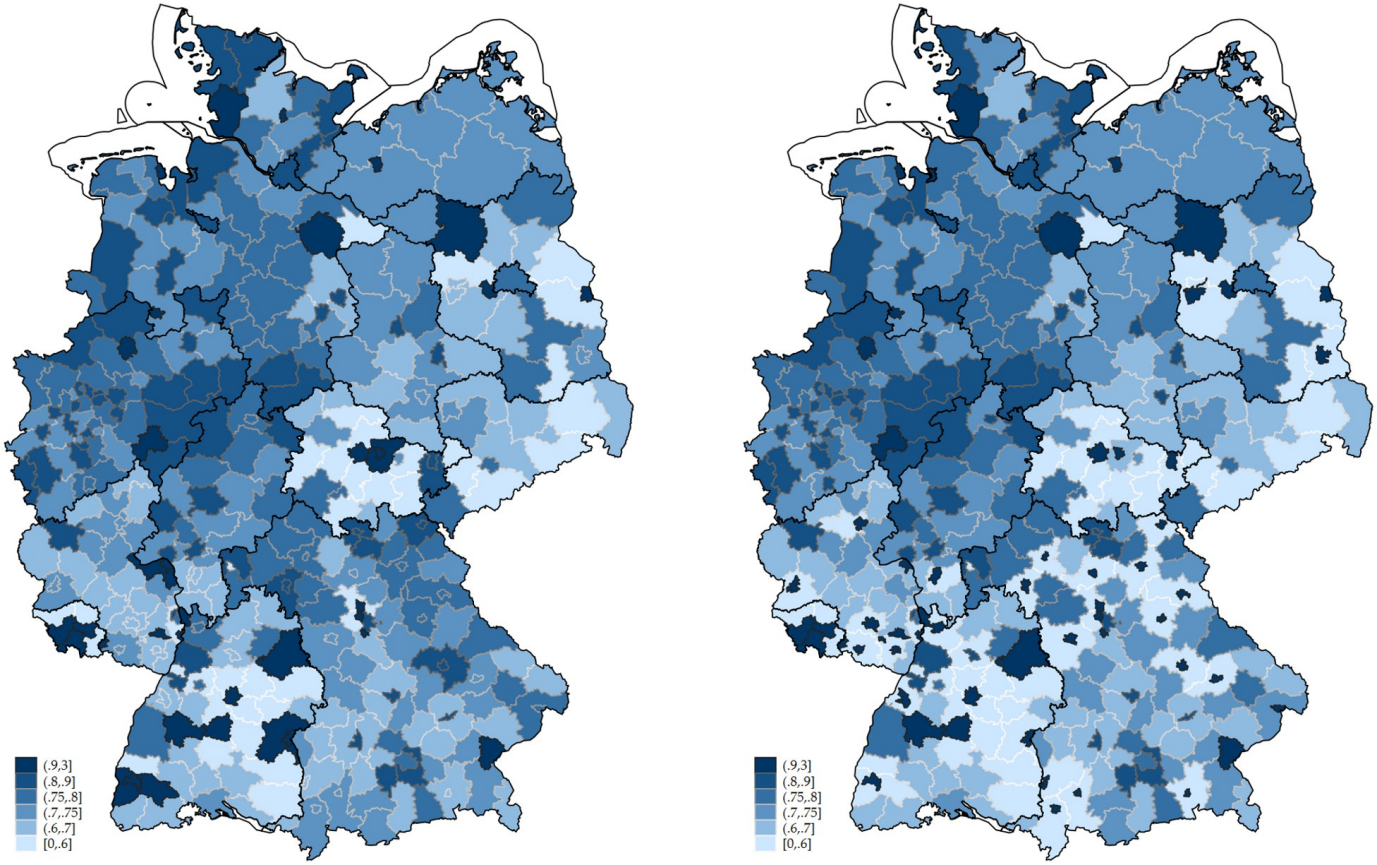
Share of 2nd vaccination per inhabitant. Left panel: Original data; Right panel: Cities merged with rural counties. Data from the Robert Koch Institute. Number of second Corona vaccinations per county until December 2022, divided by inhabitants per county. The left panel uses the original data from 400 counties. The right panel presents average numbers for 36 pairs of cities surrounded by rural counties, see text for explanations. The basemap bases on data from GeoBasis-DE / BKG (2022), dl-de/by-2–0 (www.govdata.de/dl-de/by-2-0), https://gdz.bkg.bund.de/index.php/default/digitale-geodaten/verwaltungsgebiete/verwaltungsgebiete-1-5-000-000-stand-31-12-vg5000-12-31.html.

Fig 2 shows the number of second corona vaccinations—usually defined as “complete vaccination protocol”—per inhabitant of the corresponding county until end of December 2022, calculated as follows:

$$\text{Share}\;\text{of}\;\text{second}\;\text{vaccination}\;\text{per}\;{\text{inhabitant}}_{c}=\frac{SV_{c}}{I_{c}}$$

where *c* is the county, *SV*_*c*_ is the sum of second vaccination doses carried out in county *c* until end of 2022, and *I*_*c*_ is the number of inhabitants living in county *c*. The left panel shows the map of the 400 German counties where darker colors imply larger shares of vaccinated people and lighter ones imply lower shares. A striking pattern are the small dark dots, mainly in the south of Germany. These are larger cities completely surrounded by rural counties. Often, individuals from these rural counties got their vaccination in the larger cities, leading to an overestimation of actual vaccination rates in these cities and an underestimation in the rural areas. To account for this, we manipulate the data in the following way: for the 36 cases of cities completely surrounded by one rural county, we assign both the same vaccination rate of the sum of vaccination doses divided by the sum of inhabitants in city and rural county. S1 Table in the *Supporting Information* lists all these counties. The resulting regional distribution of the share is shown in the right panel of Fig 2. In our baseline analysis, we will work with this outcome variable, that is, based on partly merging certain counties. Robustness checks with the original variable, however, yield results of similar magnitude, implying that crossing county boarders do not completely drive the results.

The mean share of individuals with two Covid vaccinations is 0.75, close to the German average reported in Fig 2, the standard deviation is 0.15. Five counties (cities) have a share larger than one while the five counties with lowest numbers have rates between 0.34 and 0.41. In robustness checks we will exclude these 10 counties. The map reveals strong variations in Corona vaccinations in Germany, both across federal states (marked by the bold borders), thereby repeating the pattern from Fig 1, as well as within states. North-West Germany has a significantly higher number of counties with larger vaccination rates than the other parts of Germany. Particularly the eastern states (Thuringia, Saxony, Brandenburg) and southern states (Baden-Wuerttemberg, Bavaria) have many counties with less than 70% vaccination coverage.

### Pool of variables on the county level

We obtain county level demographic and socio-economic variables from the INKAR database [48]. INKAR is an interactive online atlas of the Federal Institute for Research on Building, Urban Affairs and Spatial Development (BBSR), containing about 600 indicators, which enable rural-urban comparisons and analyses over the last decades. INKAR provides current regional statistics on socially important topics such as education, demography, job market, economy, accommodation, transportation, and the environment.

For the regressions, we consider those variables that are associated with vaccine uptake or hesitancy. As variations in the age structure could influence the variation in vaccinations given that older age groups were initially given preference over others when the vaccines were made available, we take into account differences in the demography by considering the age structure and also include the share of male persons, the share of non-natives, the share of people in need of long-term care, life-expectancy and fertility rate.

Previous exposure of counties to the COVID-19 pandemic might affect the willingness to vaccinate. Thus, we account for the cumulative sum of COVID-19 infections per county until December 2020, that is, at the time vaccination campaigns started. Moreover, since the spread of the COVID-19 virus is through the air, the living situation is likely to have an impact on its spread which in turn could have an influence on vaccination uptake. We, therefore, include variables that refer to the housing conditions such as the share of residential buildings, the different apartment sizes based on the number of rooms, per capita living space and the share of apartments in residential buildings.

Further potentially contributing factors to the rate of vaccine uptake are the location and its economy, education, political affiliation and healthcare accessibility. Thus, we consider the rurality, the distance to centers, net commuting and cadastral area as well as gross earnings, taxes, debts, GDP, unemployment rate and the share of people receiving minimum social security benefits, as well as the number of pupils, university students, and different educational levels of employees. In addition, we include the share of voter turnout and the votes for the different political parties and medical supply by taking into account the number of hospital beds and physicians, and the distance to the nearest pharmacy. All variables are listed in Table 1 below.

**Table 1:** Descriptives.

Variable	Description	Mean	St. dev	Min	Max
*Demographics*					
Age < 3	Share age under 3 in %	2.76	0.29	1.85	3.60
3 ≤ Age < 6	Share age 3 to under 6 in %	2.78	0.22	2.18	3.36
6 ≤ Age < 18	Share age 6 to under 18 in %	10.71	0.83	7.58	13.38
18 ≤ Age < 30	Share age 18 to under 30 in %	13.13	3.03	6.63	23.60
30 ≤ Age < 50	Share age 30 to under 50 in %	24.43	1.68	20.66	32.27
50 ≤ Age < 65	Share age 50 to under 65 in %	23.64	2.10	16.84	28.47
65 ≤ Age	Share age 65 and older in %	22.56	2.96	15.59	32.71
Women	Share of women in %	50.58	0.63	48.72	52.49
Non-native	Share of non-natives in %	10.82	5.40	2.11	36.56
Life-expectancy	Average life-expectancy of a newborn	81.15	0.97	78.28	83.92
Fertility	Total fertility rate	1.62	0.15	1.16	2.04
LTC	Share of people in need of long-term care (LTC) per 100 inhabitants	5.15	1.23	2.23	9.57
*Housing*					
COVID cases	Cumulative number of COVID cases per people until December 2020	0.02	0.01	0.00	0.05
Residential buildings	Share residential buildings with 3 or more apartments in %	16.69	10.41	3.90	49.48
1 or 2 rooms	Share apartments with 1 and 2 rooms in %	10.76	5.38	3.00	32.00
5 or more rooms	Share apartments with 5 and more rooms in %	45.35	13.44	13.58	70.34
Living space	Living space per inhabitant in m^2^	48.92	4.93	35.94	69.49
Appartm. residential buildings	Share apartments in residential buildings in %	45.91	19.27	13.96	89.41
*Area*					
Rurality	Share with population density below 150 people per km^2^	29.40	30.05	0.00	100.00
Distance major center	Avg. driving time to the nearest major center in min	23.12	16.23	0.00	69.61
Distance medium center	Avg. driving time to the nearest medium or major center in min	7.23	5.73	0.00	36.34
Net commuting	Net commuting per 100 employees (subject to social insurance) at workplace	-10.14	28.81	-143.96	61.58
Cadastral area	Cadastral area in km^2^	893.68	724.00	35.70	5495.60
*Economy*					
Gross earnings	Gross monthly earnings of employees in euros	2863.44	409.05	2244.47	5196.66
Municipal tax capacity	Municipal tax capacity in euros per inhabitant	947.34	276.42	495.01	2818.92
Business tax	Business tax in euros per inhabitant	569.97	299.05	198.99	2637.91
Sales tax	Sales tax in euros per inhabitant	90.97	37.56	34.47	309.93
Municipal debt	Municipal debt in euros per inhabitant	1490.23	1455.17	0.00	9811.02
GDP	Gross domestic product in 1,000 euros per inhabitant	38.54	16.97	16.61	188.29
Unemployment rate	Share unemployed in %	4.69	2.10	1.35	12.83
Beneficiaries SGB II	Share beneficiaries according to SGB (Sozialgesetzbuch) II aged under 65 in %	7.14	4.11	1.16	24.11
Min. social security benefits	Share receiving minimum social security benefits in %	7.27	3.64	2.13	21.98
*Education*					
Pupils	Pupils per 100 inhabitants	10.03	1.43	5.82	17.26
Students	University students per 1.000 inhabitants	28.51	52.14	0.00	379.57
No degree	Share employees without completed education in %	12.04	2.92	5.03	20.98
Academic degree	Share employees with academic education in %	12.78	5.56	5.29	35.69
Experts	Share employees who are experts in their field in %	10.70	3.87	5.14	29.34
*Politics*					
Voter turnout	Share of voters among those eligible to vote in % (2017)	75.85	3.71	64.10	84.40
CDU/CSU	Share votes for CDU/CSU (Christlich Demokratische/Soziale Union) in % (2017)	34.27	6.02	21.61	53.79
SPD	Share votes for SPD (Sozialdemokratische Partei Deutschlands) in % (2017)	20.14	6.35	7.80	38.25
Green party	Share votes for Green party in % (2017)	8.09	3.68	2.07	23.31
AfD	Share votes for AfD (Alternative für Deutschland) in % (2017)	13.38	5.33	4.94	35.46
FDP	Share votes for FDP (Freie Demokratische Partei) in % (2017)	10.11	2.48	5.11	17.65
Left-wing party	Share votes for Left-wing party in % (2017)	8.80	4.52	3.58	22.90
Other parties	Share votes for the sum of the other parties in % (2017)	5.20	1.81	2.01	12.58
*Supply medical services*					
Hospital beds	Hospital beds per 1,000 inhabitants	6.29	3.88	0.00	28.66
Physicians	Physicians per 10,000 inhabitants	14.51	4.33	7.55	30.76
Distance pharmacy	Population-weighted linear distance to the nearest pharmacy in m	1503.60	775.87	347.00	3819.00

Number of observations: 400. Source: Own calculations based on INKAR data.

### Further variables measured on the federal state level for supplementary analyses

On the individual level, different aspects and types of trust were identified as determinants of vaccination [49, 50]. Therefore, in supplementary analyses described below, we also consider four questions from the 10th wave of the European Social Survey (ESS) [51] and the German Socio-Economic Panel (SOEP) [52]—the most prominent and long-running German representative household data set. SOEP is based on about 22,000 participants and the latest wave was carried out in 2020. The German part of the ESS covers around 8,700 participants, interviews regarding the questions below took place between 2021 and 2022. Both data sets are on the individual level but only representative for the federal state, not the county level. Therefore, we use these individual level data but present averages on federal state level as suggestive complementary evidence.

We consider four questions:

- How do you see yourself: Are you generally a person who is very willing to take risks or do you try to avoid taking risks? 0 (not at all willing to take risks) to 10 (very willing to take risks). (Source: SOEP)
- Please tell me on a score of 0–10 how much you personally trust each of the institutions I read out. 0 means you do not trust an institution at all, and 10 means you have complete trust: Germany’s parliament? (Source: ESS)
- Please tell me on a score of 0–10 how much you personally trust each of the institutions I read out. 0 means you do not trust an institution at all, and 10 means you have complete trust: Scientists. (Source: ESS)
- Please tell me how much you agree or disagree with the following statement: Coronavirus is the result of deliberate and concealed efforts of some government or organization. Disagree strongly (1)—Agree strongly (5) (Source: ESS, answer categories recoded by the authors)

## Data analysis

### Basic approach

To fix ideas, consider the following linear regression model:

$$y_{c}=\beta_{0}+\beta_{1}X_{1c}+\beta_{2}X_{2c}+\beta_{3}X_{3c}+\beta_{4}X_{4c}+\beta_{5}X_{5c}+\beta_{6}X_{6c}+u_{c}$$ (1)

where *c* stands for the county. Further let:

*y*_*c*_ = COVID-19 vaccinations per inhabitant,

*X*_1*c*_ = Vector of demographic characteristics,

*X*_2*c*_ = Vector of variables reflecting previous Corona cases and housing conditions,

*X*_3*c*_ = Vector of variables reflecting area and local economy,

*X*_4*c*_ = Vector of educational levels,

*X*_5*c*_ = Vector of political preferences,

*X*_6*c*_ = Vector of supply of medical services,

*u*_*c*_ = error term.

In Section *Pool of variables on the county level* and Table 1 below, we list a number of variables for these six blocks. In total, we have a pool of 48 potential variables that may enter the regression equation. Since we do not aim to perform a causal analysis but to maximize explanatory power in a prediction sense, we use a data-driven approach to select variables from the pool of *K* = 48 variables. The approach is in the spirit of [53], here adapted to increase the explanatory power of the model. To be specific, we apply the following pre-processing procedure:

1. Start with *K* bivariate regressions of *y*_*c*_ on each of the variables from the pool of potential variables. Store the *K* values of adjusted *R*^2^.
2. Take the variable that leads to the largest adjusted *R*^2^ and put it into the set *X*_*lin*_. Let *L* be the number of variables in *X*_*lin*_. After this step, we have *L* = 1.
3. Perform *K* − *L* regressions of *y*_*c*_ on *X*_*lin*_ and each of the remaining *K* − *L* variables not in *X*_*lin*_. Store the *K* − *L* adjusted *R*^2^.
4. Take the variable that leads to the largest increase in the adjusted *R*^2^ compared to the regression from the previous step. Put in into the set *X*_*lin*_.
5. Repeat the steps 3 and 4 until no further variable increases the adjusted *R*^2^.
6. Generate all *H* possible bivariate interactions of the variables in *X*_*lin*_ after step 5. This is an additional pool of potential variables.
7. Repeat the procedure from steps 3 to 5. Now, start with *H* regressions of *y*_*c*_ on *X*_*lin*_ and one of the *H* interactions. Proceed as before where you put selected interactions into the vector *X*_*int*_ and regress *y*_*c*_ on *X*_*lin*_ and *X*_*int*_ until no further interaction increases the adjusted *R*^2^.

The regressions then use the vectors *X*_*lin*_ and *X*_*int*_. Both are composed of selected variables from the above-mentioned blocks *X*_1*c*_ to *X*_6*c*_. Allowing for many possible variables and their interaction means to have more potential variables than observations. In the data science literature, this is a common problem. Our solution to this, outlined above, is the “forward stepwise selection” discussed in Chapter 6 of [54].

### Spatial interdependencies

Note that this subsection is taken almost word by word from [38]. The error term *u*_*c*_ formulated in Eq (1) is probably not distributed independently over the individual counties. Rather, exogenous shocks in one county can be assumed to have knock-on effects in neighbouring counties. It is therefore likely that the error terms are correlated with each other spatially. To take this into account, we formulate the error term as

$$u_{c}=\rho \sum_{j=1}^{400}m_{cj}u_{j}+\varepsilon_{c}$$ (2)

where *m*_*cj*_ represents the weighting factor for the effect of a shock in county *j* on county *c*. Formulated more compactly, we get *u* = *ρMu* + *ε* where *u* is the vector of all error terms (dimension 400 × 1), *M* is the weighting matrix (400 × 400) and *ε* the vector of errors whose elements are assumed not to correlate with each other. The lower the spatial distance between two counties, the stronger the correlation of the error terms should be. In the definition of the weighting matrix this is achieved by the fact that the weights for adjacent counties are greater than for more distant ones. For *M*, we use an *inverse distance matrix*, in which the inverse of the distances between the county centroids are used as elements and the main diagonal contains zeros. The shape files of the counties from the Federal Agency for Cartography and Geodesy (as of 1.1.2022) were used to calculate the distances and transferred into Stata format with the help of the Stata programme shp2dta. We follow [55] and normalize the elements with the eigenvalue standardisation, in which every element is divided by the largest module of the eigenvalues of *M* (see [56], for a discussion of the normalisation procedures).

Furthermore, it can be assumed that the vaccination rates themselves are also subject to spatial interdependencies. It is conceivable, for example, that local vaccination initiatives have positive spill over effects from one county to another. Moreover, the issue of differences between vaccination location and home county may lead to negative spatial interdependence in the outcome variable. In a more general model in the tradition of [57]’s spatial model or the expanded spatial autoregressive model with spatial autoregressive disturbances (SARAR, [58]), the following variant is estimated:

y=λWy+Xβ+u (3)

Here *W* is again a weighting matrix. We define the matrix *W* as a *contiguity matrix* where only the contingent counties are taken into account and the weights for all others are set at 0. To estimate the parameters *β*, *ρ* und λ we use the Stata module *sppack*, which contains both the estimator for the model formulated above (spreg) and the programme for creating the weighting matrix (see [59]).

To be specific, we run the following spatial regression model:

$$y_{c}=\lambda Wy+\beta_{0}+\beta_{1}X_{c,lin}+\beta_{2}X_{c,int}+u_{c}$$ (4)

where

$$u_{c}=\rho \sum_{j=1}^{400}m_{cj}u_{j}+\varepsilon_{c}$$

This spatial regression model is also used for the variable selection described above.

### A compact approach to present the results

For an easier understanding of how much of the differences between federal states can be explained, we then turn to a more compact approach. We perform a step-wise regression. We start with a regression on a constant only, go on with a regression on a constant and variables from vector *X*_1*c*_, then add *X*_2*c*_, and so on. All regressions follow the spatial-econometric approach of Eq (2) and the variable selection procedure. However, when using block *X*_1*c*_ only, only variables from this block are in the pool of potential variables. The same applies when block *X*_1*c*_ and *X*_2*c*_ are used and so on. After each regression, we store the residuals and calculate the federal state averages of the residuals. The standard deviation of these average residuals should be smaller the larger the explanatory power of our model is. The results are presented in Table 3.

## Results

### Baseline results

Table 1 provides the descriptive statistics of the pool of variables on the level of the 400 counties. Table 2 shows the regression results of the model with the highest predictive power according to the spatial regression model outlined in Section *Spatial interdependencies* and the variable selection procedure outlined in Section *Basic approach*. The table includes the chosen variables and interactions. Both *ρ* and λ are negative. This most likely reflects the definition of the outcome variable, capturing vaccinations where they took place, not where individuals live. This possibly induces a negative correlation in vaccination rates across neighboring counties. The spatial model takes this into account. However, *ρ* is not significantly different from zero and λ is small in magnitude. All in all, spatial correlations do not seem to play a large role in this application.

**Table 2:** Regression results.

	Coef.	std. err
Age < 3	0.166	(0.31)
6 ≤ Age < 18	-1.939***	(0.75)
65 ≤ Age	0.146***	(0.05)
Women	0.160	(0.15)
Life-expectancy	-0.225**	(0.10)
COVID cases	2.857**	(1.17)
Rurality	-0.113***	(0.03)
Distance major center	0.029	(0.04)
Distance medium center	0.658***	(0.18)
Cadastral area	0.000**	(0.00)
Gross earnings	0.005***	(0.00)
Sales tax	-0.014**	(0.01)
Municipal debt	-0.000	(0.00)
Pupils	1.140***	(0.43)
Students	-0.039*	(0.02)
Academic degree	0.660***	(0.25)
Experts	-0.784**	(0.35)
AfD	-0.656***	(0.21)
AfD × Experts	-0.004**	(0.00)
AfD × Rurality	0.000**	(0.00)
AfD × 6 ≤ Age < 18	0.011***	(0.00)
AfD × Students	0.000*	(0.00)
AfD × Academic degree	0.004***	(0.00)
AfD × Municipal debt	-0.000	(0.00)
AfD × Women	0.011***	(0.00)
AfD × Sales tax	0.000***	(0.00)
AfD × Gross earnings	-0.000**	(0.00)
Experts × Cadastral area	-0.000	(0.00)
Experts × Pupils	0.002	(0.00)
Experts × Age < 3	0.131*	(0.07)
Experts × Academic degree	-0.000	(0.00)
Experts × Municipal debt	-0.000***	(0.00)
Experts × 65 ≤ Age	0.021***	(0.01)
Experts × COVID cases	0.001	(0.00)
Experts × Gross earnings	0.000	(0.00)
Life-expectancy × Rurality	0.001***	(0.00)
Life-expectancy × Distance medium center	-0.006***	(0.00)
Life-expectancy × 6 ≤ Age < 18	0.029***	(0.01)
Life-expectancy × Municipal debt	0.000	(0.00)
Life-expectancy × Gross earnings	-0.000*	(0.00)
Distance major center × Rurality	0.000	(0.00)
Distance major center × Cadastral area	0.000	(0.00)
Distance major center × Women	-0.001	(0.00)
Distance major center × COVID cases	0.002***	(0.00)
Rurality × 6 ≤ Age < 18	0.001	(0.00)
Rurality × Age < 3	-0.002	(0.00)
Rurality × Academic degree	-0.000**	(0.00)
Rurality × Municipal debt	-0.000	(0.00)
Rurality × 65 ≤ Age	0.000	(0.00)
Rurality × COVID cases	-0.001**	(0.00)
Rurality × Gross earnings	0.000***	(0.00)
Distance medium center × Pupils	-0.003	(0.00)
Distance medium center × 6 ≤ Age < 18	-0.003	(0.00)
Distance medium center × Students	0.000***	(0.00)
Distance medium center × 65 ≤ Age	-0.003**	(0.00)
Distance medium center × Gross earnings	-0.000**	(0.00)
Cadastral area × 6 ≤ Age < 18	-0.000***	(0.00)
Cadastral area × Age < 3	0.000**	(0.00)
Cadastral area × Students	-0.000*	(0.00)
Cadastral area × COVID cases	0.000	(0.00)
Cadastral area × Sales tax	0.000	(0.00)
Pupils × Students	0.000*	(0.00)
Pupils × Municipal debt	0.000***	(0.00)
Pupils × Women	-0.024***	(0.01)
Pupils × 65 ≤ Age	0.001	(0.00)
6 ≤ Age < 18 × Municipal debt	-0.000**	(0.00)
6 ≤ Age < 18 × COVID cases	-0.105***	(0.02)
6 ≤ Age < 18 × Sales tax	0.001**	(0.00)
6 ≤ Age < 18 × Gross earnings	-0.000*	(0.00)
Age < 3 × Students	-0.001	(0.00)
Age < 3 × Academic degree	-0.150***	(0.05)
Age < 3 × Sales tax	0.002	(0.00)
Students × Municipal debt	0.000***	(0.00)
Students × Women	0.001**	(0.00)
Students × COVID cases	-0.001***	(0.00)
Students × Sales tax	0.000	(0.00)
Academic degree × Women	0.002	(0.00)
Academic degree × 65 ≤ Age	-0.018***	(0.01)
Municipal debt × COVID cases	0.000***	(0.00)
Municipal debt × Gross earnings	0.000*	(0.00)
Women × COVID cases	-0.026	(0.02)
65 ≤ Age × COVID cases	-0.017***	(0.01)
65 ≤ Age × Gross earnings	-0.000***	(0.00)
COVID cases × Sales tax	-0.001*	(0.00)
Sales tax × Gross earnings	-0.000***	(0.00)
λ	-0.063**	(0.03)
*ρ*	-0.173	(0.17)
*R* ^2^	0.52	

Number of observations: 400.

* *p* < 0.10,

** *p* < 0.05,

*** *p* < 0.01

Note that Table 2 reports coefficients but not marginal effects of certain characteristics. The many interactions make the interpretations of the coefficients complicated. However, many of the baseline coefficients (though not all) go into the expected directions. For instance, the share of individuals over 65 years and previous exposure to the COVID pandemic (measured by COVID cases) have a positive baseline coefficient, while the share of voters for right populist parties (AfD) has a negative coefficient. A higher share of individuals with academic degree goes along with more vaccinations, indicating a positive relationship between education and vaccination. The share of women is positively associated with vaccination rates but the relationship is not statistically significant. All in all, with an *R*^2^ of 0.52 we can explain more than half of the variation on county level using variables on the socio-demographic structure.

Table 3 reports the results of the approach outlined in Section *A compact approach to present the results* where we stepwise include variables in order to understand their share of explained variation in Corona vaccinations. A regression of the COVID vaccinations on only demographics has an *R*^2^ of 19% and can reduce the standard deviation of the residuals by 48% at the federal state level. Previous COVID exposure and housing increases the share to 54%. Variables for area and economy increase it further to 65%. Education, politics and supply of medical services do not add much, once the other variables are controlled for, but ultimately 52% of the variation on county level and 72% on federal state level can be explained by our observable characteristics. Of course, these single numbers also depend on the ordering of the blocks but the total number after all blocks are included is invariant to the ordering.

**Table 3:** Reduction in variation.

Adjustment	R^2^ (%)	State level	
		Std. dev.	Reduction (%)
None	0	.036	0
… + Demographics	19	.019	48
… + Cases and Housing	24	.016	54
… + Area and Economy	27	.013	65
… + Education	48	.01	72
… + Politics	52	.01	72
… + Supply medical services	52	.01	72

### Robustness checks

Table 4 reports results from robustness checks. These follow the same estimation protocol as above but do not split up the results by different blocks of variables and merely show results for all blocks *X*_1*c*_ to *X*_6*c*_. We first run the regressions with 390 observations where the ten counties with largest and smallest vaccination rates are dropped. We cannot rule out that these are still strongly affected by differences in vaccination location and place of residence. Moreover, we use the original RKI data without merging numbers for 36 county-pairs (see Section *Data*). The results are basically the same when we drop the 10 outliers. Using the original data, we receive a very large *R*^2^ of 84. However, we tend to have more trust in the baseline specification.

**Table 4:** Robustness checks.

Sample	R^2^ (%)	Reduction (%) Federal state level
Baseline	52	72
Without 10 outliers	50	78
Original data	84	79

### Potential remaining factors measured at the federal state level

The variables used in the previous analysis cannot fully explain the regional variation in differences in COVID-19 vaccination. Therefore, this subsection provides examples of variables that can be expected to be associated with vaccination but for which data are not available at county level.

**Fig 3:**
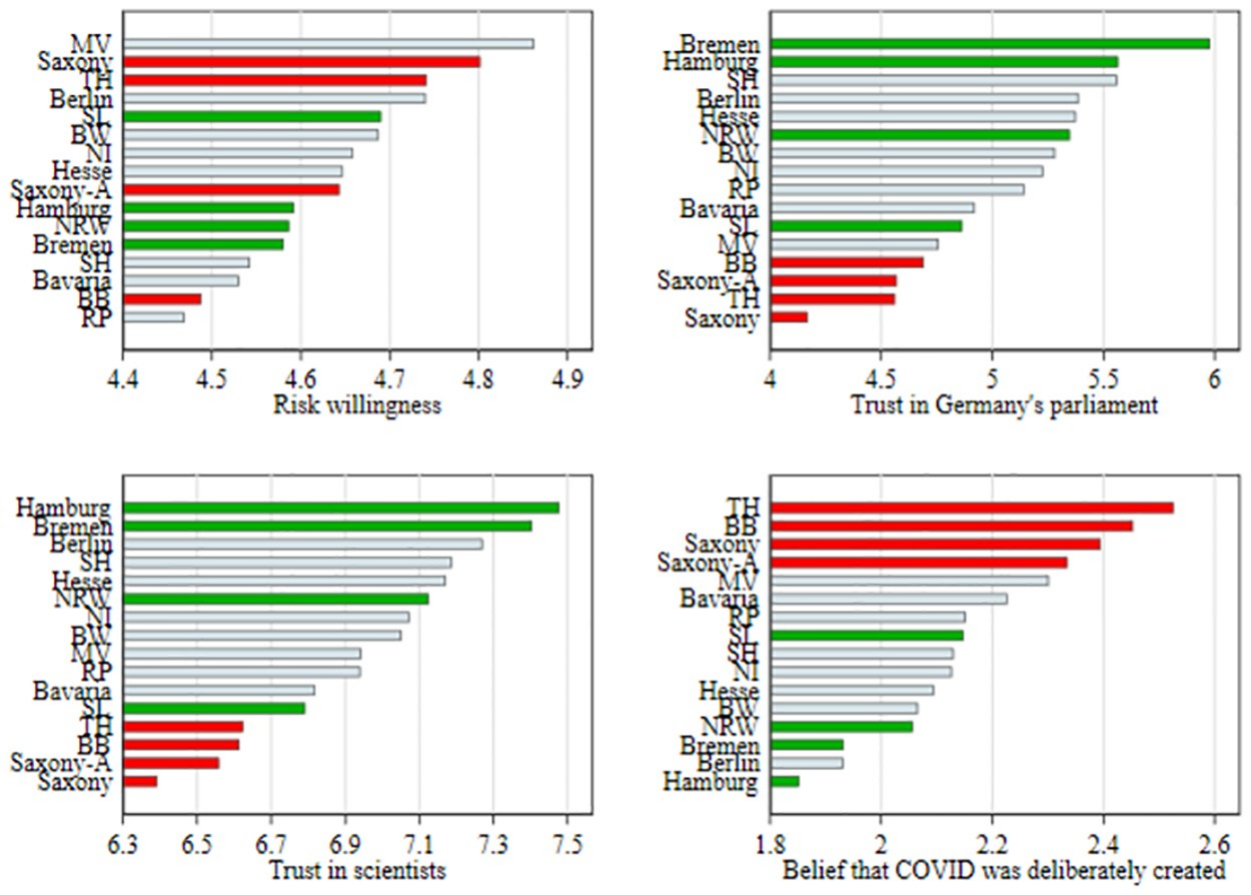
Federal state-level differences in risk preferences and selected statements. Sample means by federal states presented. MV stands for Mecklenburg-West Pomerania, TH = Thuringia, BB = Brandenburg, RP = Rhineland Palatinate, SL = Saarland, NI = Lower Saxony, SH = Schleswig-Holstein, Saxony-A. = Saxony-Anhalt, NRW = North Rhine-Westphalia, BW = Baden-Wuerttemberg. Upper left panel: *Source:* SOEP; Other panels: *Source:* ESS.

Fig 3 reports the sample means in the different federal states of the four answers concerning risk willingness, trust in Germany’s parliament, trust in scientists and the belief that COVID was deliberately created. For the sake of presentation, we color the number of the four states with highest vaccination rates (see Fig 1) in green and the four states with the lowest vaccination rates in red. Specifically, the four states with lowest vaccination rates also form a consistent group of highest or lowest ranked states in these four panels (with one exception). In these states, individuals tend to be more risk loving, have less trust in the German parliament and scientists in general, and are more likely to believe that the Corona virus was deliberately created. Bavaria and Mecklenburg-West Pomerania, the states that rank 5 and 6 from the bottom of vaccination status, also broadly rank in this range in the four answers. It is less consistent with the states with highest vaccination rates. By and large, however, they rank on the other extreme also in these four questions. Hence, Fig 3 offers additional explanatory factors for regional variations in vaccination rates.

## Discussion

In terms of Corona vaccination rates, Germany ranks only in the midfield among the industrialized countries. This is partly due to some regions within Germany that have very low vaccination rates. In this study we seeked to understand the determinants of the regional variation in Corona vaccination in Germany. It turned out that differences in demographic status are able to explain a considerable share of this regional variation. For instance, regions with a higher share of individuals older than 65 have higher vaccination rates. All in all, differences in demographics alone account for 48 percent of the variation in Corona vaccination on the federal states level. Arguably, differences in vaccination rates due to differences in demographics seem to be justified, for instance given that older individuals belong to the high-risk group and should have higher vaccination rates. Thus, parts of the regional differences observed in Figs 1 and 2 are something policy does not need to worry about or even address. Yet, this refers to only half of the regional variations.

Using a host of other characteristics together with a highly flexible spatial-econometric model, we came to the result that around 45% of the county-level differences and 72% of the federal state-level differences can be explained by observable characteristics. Important factors other than demographics are previous exposure to Corona in the county, the economic situation and educational status. A number of studies have reported positive correlations of education and vaccination on the individual level (e.g., [14]), thus, this does not come as a surprise. Our explained share is lower than in studies from the USA, where, based on the adjusted *R*^2^, about 76–79% could be explained [23, 24]. Apparently, our results are not directly comparable to these studies as they use other data, other methods and refer to a different health care system. Nevertheless, an important share of variation in vaccination rates among German counties is left unexplained by basic “hard” factors. Thus, in the second part of the analysis we turned to “soft” factors.

We found suggestive evidence that preferences like risk attitudes and trust might be important explanatory factors for these differences. In federal states with low vaccination rates, individuals in two representative surveys were also more likely to report higher levels of willingness to take risks and lower levels of trust in science. While differences in vaccination due to risk preferences seem justified (when being unvaccinated is just an expression of the preference to take risks), policy should take mistrust in science seriously.

That a person’s trust in institutions or authorities is an important factor in their intention to vaccinate was found in many studies [49, 60, 61]). For [62], trust as part of social capital is a plausible channel through which the Communist past of the East German countries affects vaccination willingness. The negative correlation between belief in conspiracy theories and vaccination willingness has also already been confirmed in empirical studies on the individual level. For Germany, [63] find that parents who have high levels of agreement with vaccine conspiracy theories show a lower willingness to get vaccinated against the corona virus. [64] find for Turkey and the UK that people who believe in a natural origin of the Corona virus are more likely to be vaccinated against the virus.

Trust and belief in conspiracy theories are also important factors in predicting other kinds of preventive behavior: People with a higher trust in government are more likely to comply to the recommended health behaviors such as handwashing and self quarantine [65] and people who believe in conspiracy theories related to COVID are less likely to adhere to measures recommended to contain the Corona virus such as wearing masks. Moreover, they are less likely to intend to get vaccinated [66]. It should be noted, however, that we are not aware of studies that explain regional variations in other preventive measures. Thus, on the one hand, our results are not directly comparable. On the other hand, the strong correlation between vaccination and other preventive measures allows the prediction that regional variations in other measures may share similarities with regional variations in vaccination.

Thus, improving the trust in science and the government as well as the fight against conspiracy theories in general might not only be important in itself to safeguard democracy, but moreover be effective tools to improve vaccination rates. This includes the transparent and impartial discussion and evaluation of all measures taken to fight the COVID pandemic (such as vaccination campaigns, compulsory vaccination in the health care sector, compulsory mask wearing, school closures and lockdowns). An examination with an open mind and the courage to tell unpleasant truths (e.g., where scientist made recommendations regarding the fight of the Corona crisis that turned out to be wrong in hindsight) may be a way to increase trust in science again. This will be particularly relevant for future pandemics.

There are several limitations to our study. Our study design is not able to provide any causal evidence and, thus, does not allow to immediately derive clear policy recommendations and all conclusions derived here must be taken with caution. The main data limitation of this study is that vaccination rates are measured in the county where vaccinations are carried out and not where individuals live. This may lead to underestimations in some areas and overestimations in others. A large part of differences in vaccination rates remains unexplained. Access to more and different variables at the county level might further reduce the unexplained share. Finally, more granular data on a level below county level should be helpful, also to avoid potential problems of ecological fallacy. To date these data do not exist for Germany.

## Conclusion

High vaccination rates have repeatedly been stressed by many scholars as a decisive factor to fight pandemics such as the COVID-19 pandemic. However, vaccination rates differ strongly across and within countries where some regions have vaccination rates that are generally perceived too low. In this study we analyzed regional variations of COVID-19 vaccination and their determinants in Germany. A highly flexible spatial-econometric model came to the result that around 52% of the county-level differences and 72% of the federal state-level differences can be explained by different county structure such as demographics, housing, economy, education, and party preferences. Around 50% of the differences on federal state level are due to differences in demographics. Thus, arguably, up to 50% of the regional variations may be regarded as justified. However, in future pandemics, policymakers may want to reduce the other 50%. We find suggestive evidence that soft factors on preferences like risk attitudes and trust might be important additional explanatory factors. Based on our findings we recommend improving the trust in science and the fight against conspiracy theories as possibly effective tools to improve vaccination rates and effectively fight pandemics.

## Supporting information

S1 Table Counties assigned the average vaccination rate. (PDF)
