# Supplementary material for: Regional variations in vaccination against COVID-19 in Germany
Source: PLoS One. 2024 Apr 18;19(4):e0296976. doi: 10.1371/journal.pone.0296976 (PMC11025766; doi:10.1371/journal.pone.0296976)
Supplement: S1 Table — (PDF) [file pone.0296976.s001.pdf]

S1 Table. Counties assigned the average vaccination rate

**Name county 1/ name county 2 (county id 1/county id 2)**
Kempten/Oberallgäu (9763/9780)
Kaufbeuren/Ostallgäu (9762/9777)
Rosenheim/Rosenheim (9163/9187)
Straubing/Straubing-Bogen (9263/9278)
Ansbach/Ansbach (9561/9571)
Regensburg/Regensburg (9362/9375)
Amberg/Amberg-Sulzbach (9361/9371)
Weiden in der Oberpfalz/Neustadt an der Waldnaab (9363/9374)
Bayreuth/Bayreuth (9462/9472)
Hof/Hof (9464 /9475)
Bamberg/Bamberg (9461/9471)
Schweinfurt/Schweinfurt (9662/9678)
Würzburg/Würzburg (9663/9679)
Passau/Passau (9262/9275)
Weimar/Weimarer Land (16055/16071)
Gera/Greiz (16052/16076)
Cottbus/Spree-Neiße (12052/12071)
Brandenburg an der Havel/Potsdam-Mittelmark (12051/12069)
Trier/Trier-Saarburg (7211/7235)
Pirmasens/Südwestpfalz (7317/7340)
Landau in der Pfalz/Südliche Weinstraße (7313/7337)
Kaiserslautern/Kaiserslautern (7312/7335)
Mainz/Mainz-Bingen (7315/7339)
Flensburg/Schleswig-Flensburg (1001/1059)
Bremen/Bremerhaven/Cuxhaven (4011/4012/3352)
Darmstadt/Darmstadt-Dieburg (6411/6432)
Heidelberg/Rhein-Neckar-Kreis (8221/8226)
Baden-Baden/Rastatt (8211/8216)
Freiburg im Breisgau/Breisgau-Hochschwarzwald (8311/8315)
Memmingen/Unterallgäu (9764/9778)
Ulm/Alb-Donau-Kreis (8421/8425)
Koblenz/Mayen-Koblenz (7111/7137)
Heilbronn/Heilbronn (8121/8125)
Halle/Saale (15002/15088)
Bonn/Rhein-Sieg-Kreis (5314/5382)
Kassel/Kassel (6611/6633)
